# D-Track—A semi-automatic 3D video-tracking technique to analyse movements and routines of aquatic animals with application to captive dolphins

**DOI:** 10.1371/journal.pone.0201614

**Published:** 2018-08-16

**Authors:** Patrícia Rachinas-Lopes, Ricardo Ribeiro, Manuel E. dos Santos, Rui M. Costa

**Affiliations:** 1 Champalimaud Neuroscience Programme, Champalimaud Center for the Unknown, Lisboa, Portugal; 2 MARE – Marine and Environmental Sciences Centre, ISPA – Instituto Universitário, Lisboa, Portugal; Imperial College London, UNITED KINGDOM

## Abstract

Scoring and tracking animal movements manually is a time consuming and subjective process, susceptible to errors due to fatigue. Automated and semi-automated video-based tracking methods have been developed to overcome the errors and biases of manual analyses. In this manuscript we present D-Track, an open-source semi-automatic tracking system able to quantify the 3D trajectories of dolphins, non-invasively, in the water. This software produces a three-dimensional reconstruction of the pool and tracks the animal at different depths, using standard cameras. D-Track allows the determination of spatial preferences of the animals, their speed and its variations, and the identification of behavioural routines. We tested the system with two captive dolphins during different periods of the day. Both animals spent around 85% of the time at the surface of the Deep Area of their pool (5-meters depth). Both dolphins showed a stable average speed throughout 31 sessions, with slow speeds predominant (maximum 1.7 ms^-1^). Circular swimming was highly variable, with significant differences in the size and duration of the “circles”, between animals, within-animals and across sessions. The D-Track system is a novel tool to study the behaviour of aquatic animals, and it represents a convenient and inexpensive solution for laboratories and marine parks to monitor the preferences and routines of their animals.

## Introduction

Ethologists and other behavioural scientists have traditionally scored animal movements in experimental settings by hand. This low cost, traditional technique [1–3], has the drawback of being a very time consuming process and susceptible to errors due to fatigue, drift and subjectivity [3–4]. To overcome these problems, automated video-based tracking methods have been developed over the years in controlled environments to explore and extract the detailed information that is contained in videos of moving animals (e.g., Kabra *et al*. studies [5]). Due to the high temporal and spatial resolution of these techniques, it is now possible to perform the tracking of different animals and transform the video data into trajectories of positions over time [5–6].

These techniques are now widely used with commercial video analysis systems in a variety of experiments [7–10], with different animals such as *Caenorhabditis elegans* [11–13], fish [6, 14–16], insects [6, 17–18], rodents [6, 19–23], primates [24] and pigs [3, 25]. However, the studies mentioned above have been mostly limited to small cages and restricted environments, as noted by Ballesta *et al*. [24]. Previous developments seemed less suited to track the movements of large aquatic animals such as bottlenose dolphins in their sizable pools.

To overcome the obstacles to animal tracking in a large pool with varying depths, we developed D-Track, a software that tracks aquatic animals in their habitat, without disturbance. This novel methodology works with at least two cameras, positioned at opposite locations and different heights, and not only from top or lateral views (as in most existing systems). It combines the information of specific measurements of the pool with the images of multiple cameras to reconstruct a 3D environment. It is also prepared to perform the analysis with different light and weather conditions, and it tracks the animals at different depths. The tracking is semi-automatic and does not suffer from propagation of errors, requiring only some adjustments related to the sunlight and wind levels at the site. D-track was designed to be adaptable to other captive scenarios and it needs no special modifications for different species. It represents an improvement that may allow marine parks and laboratories to easily be aware of behavioural or movement anomalies with their animals without spending too much effort or time.

## Materials and methods

### Subjects and facility

The subjects of this study were two captive common bottlenose dolphins (*Tursiops truncatus*) held at the marine theme park Zoomarine, located in the Algarve, a Southern region of Portugal. Both animals were adult males: HM5 (born in 1995 and with 185 Kg, 2.46 m) and AM4 (born in 2004 and with 172 Kg, 2.55 m). At the time of this study, these dolphins were housed in a covered pool (Lagoa Azul).

**Fig 1:**
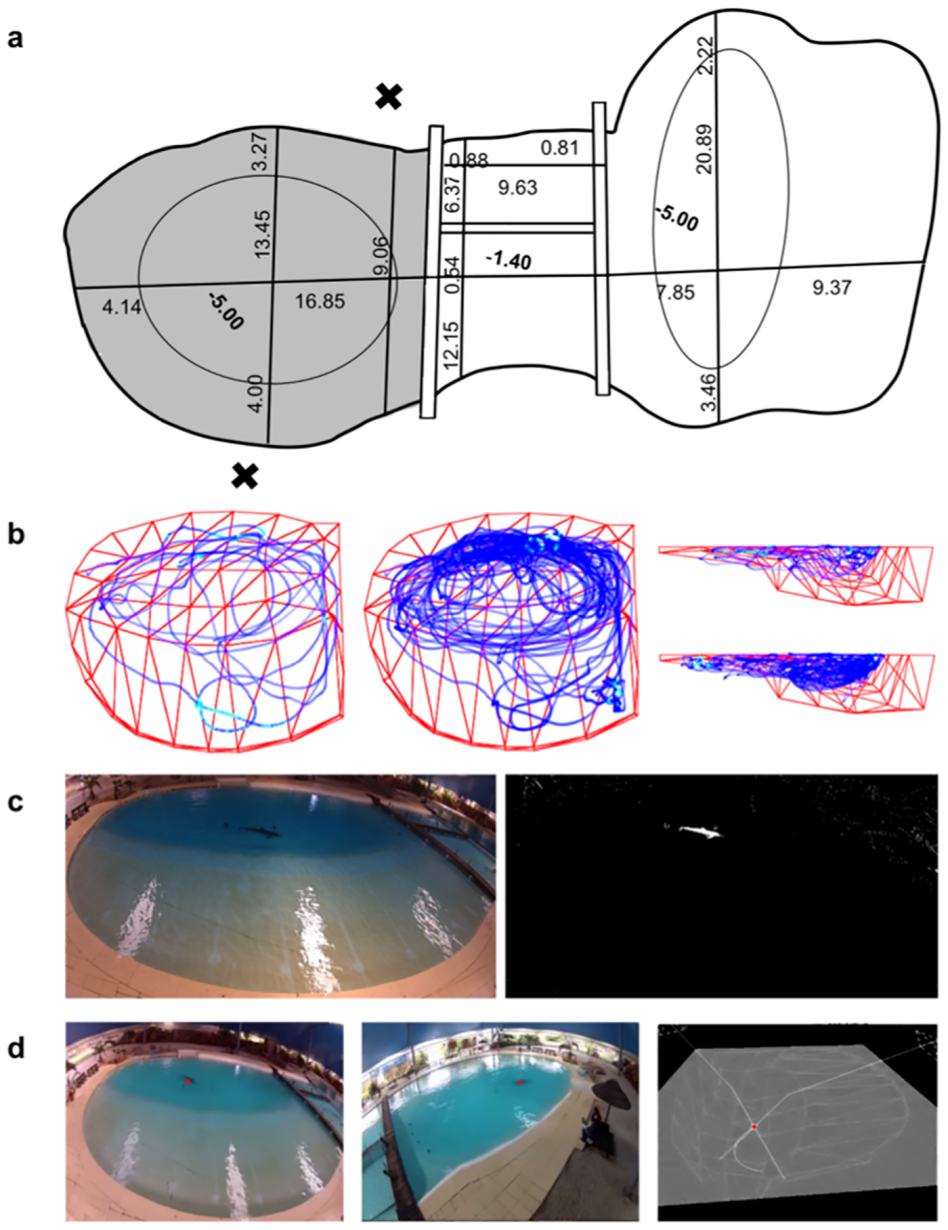
Entire pool with dimensions and sample trajectories. (a) Diagram of the experimental pool. Black X marks show the position of the two video cameras, with 170° lenses at approximately 4-m high. (b) Example of the trajectory of the focal dolphin in the pool, after 5 and 30 minutes of a session, from above and laterally. (c) An example of a video frame recorded with the focal animal (left), and with the background subtracted for analysis (right). The threshold had to be defined manually since it depends on the weather conditions. (d) Images showing the dolphin, with the red dot from the tracking recognition, and with the three-dimensional reconstruction of the pool. The intersection of the two vectors from the cameras to the dolphin determine the 3D position of the animal in the pool.

The complete pool, 47-m long, is divided into 4 smaller sections that are separated by perforated fiberglass doors and panels (Fig 1a).

The animals were video recorded in a pool section (the experimental pool), with 21-m diameter and a maximum depth of 5 m. The animals usually spent time in all pool sections. The subjects in the experimental pool were separated from the remaining group by fiberglass doors, with visual and acoustic contact among them.

Two cameras (GoPro Hero 2; GoPro, Inc.), with 170° lenses, 1280x720 pixel resolution and 30 frames per second video, paired with a Smart Remote Control, were mounted in opposite locations in the pool.

The facility where the study was conducted (Zoomarine Algarve, of Mundo Aquático S.A.) is accredited by, and follows the standards and guidelines for husbandry and scientific research of the Alliance of Marine Mammal Parks & Aquariums, the European Association for Aquatic Mammals and the European Association of Zoos and Aquaria. Careful procedures were implemented to minimize disturbance to the animals in this study, who were only observed and not manipulated. The project was approved by Zoomarine’s Board and all technical Directors (including the Curator, Veterinarian Director and Scientific Director).

### Data collection

Behavioural data collection was conducted between November 2012 and April 2014. Video recordings of single animals in the experimental pool (either HM5 or AM4) were obtained through 31 sessions, varying from 20 to 60 minutes duration, between 9 AM and 6 PM, totalizing 18 hours of video recordings. These video recordings were always produced in the presence of trainers, although with minimal interactions between trainers and animals. The set up of the equipment was prepared at least 10 minutes before the data collection session, to minimize the animal’s reactivity.

The trainers’ routines around the different areas of the pool, such as feeding and training sessions with other animals in other sections of the pool, were carried out normally.

### Calibration of the cameras

Due to wide-angle lenses, the cameras covered the entire experimental pool, adding, however, a fish-eye effect that distorted the image. Consequently a calibration to match the pixels coordinates to the correct position of the animal, as well as the size was needed. The calibration was made according to Carstensen’s, 2001 [26]. The following camera parameters, matrix (1) and matrix (2) were used:

Distortionmatrix=[−0.335050702095,0.120226070285,0.0,0.0,−0.0201695654541] (1)

$$\begin{array}{lll} Intrinsic\;parameters\;matrix & = & \left[[654.55,0,1279/2]\right]\\ & & \left[0,654.55,719/2\right]\\ & & \left[0,0,1]\right] \end{array}$$ (2)

### Overview of the system

To obtain the 3D data, two synchronized video cameras, recording in 32 GB SD card and .mp4 format, were fixed in approximately opposite locations to enable the recording of the entire habitat from two different perspectives. Information about the position and orientation of the cameras was used to calculate the dolphin’s 3D position. Therefore, detailed measurements of the pool tiles and specific distances between objects in the area were included in the D-Track software, where five points, visible from both cameras, were chosen as input calibration points to the RANSAC Perspective-*n*-Point algorithm [27].

The images of both cameras were then merged to reconstruct the 3D scenario.

### 3D Data acquisition

To apply the colour filters to the video frames and to estimate the 3D features of the scenario a Python 3 script (Python Software Foundation) with OpenCV 3.0 library was used (Fig 1c).

The centroid of the moving dolphin, in pixels, corresponds to a vector originated from the focal point of the camera to the subject in the pool. The intersection of the two vectors, one from each camera, is used to calculate the 3D position of the animal in the pool (Fig 1d).

To find the vectors for both cameras, the Eqs (3) and (4), from the pinhole camera model, were used:

$$\begin{array}{c} x^{\prime }=x/z,y^{\prime }=y/z,u=fx*x^{\prime }+cx,v=fy*y^{\prime }+cy\\ ⬄ \end{array}$$ (3)

x=(u−cx)*z/fx,y=(v−cy)*z/fy (4)

where *u* and *v* are the coordinates of the projection points in pixels; *cx* and *cy* is the main point usually at the centre of the image; and f_x_ and f_y_ are the focal lengths expressed in pixel units.

To obtain the vector we calculated the points for z = 0 and z = 1.

### Dolphin tracking

Image-subtraction techniques were used to detect the moving subject through the contrast that separates the animal from the background (Fig 1c). The tracking algorithm applies a mask to each frame that neglects the regions outside the tracking area. Several imaging filters, such as an adaptive threshold, colour ranges filtering, erosion and dilation, were used to extract the subject’s blob and to remove the remaining noise. The parameters for these filters had to be defined manually since it depends on the natural light in the scenario and the visibility of the subject.

After applying the filters, the pixel coordinates corresponding to the centroid of the dolphin in each frame is extracted. Subsequently, a Gaussian filter (sigma = 32) is applied to all the resulting coordinates to smooth the data and eliminate some centroid jittering, correcting any tracking errors. Based on the depth and specific measurements of known distances, the water refraction index of 1.4 was estimated and applied. The intersection of both subject’s centroids gives the 3D position of the dolphin (Fig 1d).

To avoid artifacts caused by tracking inaccuracy, an outlier analysis was made calculating the median, lower quartile, upper quartile, interquartile range, inner and outer fences of the data. The major outliers were discarded.

### Quantification of the position of the dolphin in the pool

D-Track permits a variety of analyses, including trajectories, pool occupancy/preferences, speed and routines of the animals in the pool.

To study the dolphin’s 3D trajectories and routines in the pool throughout sessions, the animal’s position in each frame is extracted. Plotting the positions of the animals through time enables the analysis of the entire trajectory and routines of the animals.

To analyse the occupancy/preferences of the animals in the pool it was necessary to isolate the corresponding portion of the arena to be quantified. The frames associated with the maximum depth area were labelled as Deep Area at the surface of the pool. The difference between the total frames and those corresponding to the Deep Area were designated the Shallow Area of the pool. To study the preferences of both animals in relation to the deepest part of the pool (close to the 5 meters depth), the area below the maximum depth of the Shallow Area (approximately 1.4 meters depth) was designated as Bottom.

The speed of the animals was calculated through the difference between 3D positions of the dolphin in two consecutive frames (positionframe_index−position_frame_index-1_), which results in distance moved between each two frames (33.33 milliseconds). The dolphin’s maximum speed was considered the highest speed value registered and was transformed in meters per second. This value was then divided into three categories of speed based on tertiles: low (from 0 to 1.7 ms^-1^), intermediate (from 1.7 to 3.4 ms^-1^) and high speed (from 3.4 to 5.1 ms^-1^).

To study the routines of the animals, the trajectories from all sessions were plotted for both animals. Important occurrences were analysed in this section: “swimming ring” sizes (complete circles around the pool), the influence of trainers’ presence and interactions of the focal animal with dolphins in adjacent pools. To study the variation in the swimming ring sizes, the start and end positions of each ring were extracted, and the distance and speed between both points was calculated. To study the influence of the trainers’ presence in the routines of the focal animals, as well as the interaction between the subject and other dolphins in the adjacent pool, the corresponding section of the scenario was isolated and the frames extracted.

### Statistical analysis

The Kruskal-Wallis test was used to examine differences in the size and duration of the swimming rings across HM5 and AM4 and between sessions of each subject.

All statistical analyses were performed using *IBM SPSS Statistics 21* (IBM Inc.).

## Results

D-Track is a software that tracks dolphin positions in a pool, using multiple digital video cameras (Fig 1b).

To test the potential for semi-automated tracking analysis, we quantified the occupancy/preferences of two adult animals in the pool (labelled HM5 and AM4), their instantaneous speeds and circular swimming paths. The D-track software (http://d-track.readthedocs.io), written in Python detects the moving objects, frame by frame, and so the focal dolphin in the pool is successfully tracked (Fig 1d).

### Quantification of the dolphin positions in the pool

**Fig 2:**
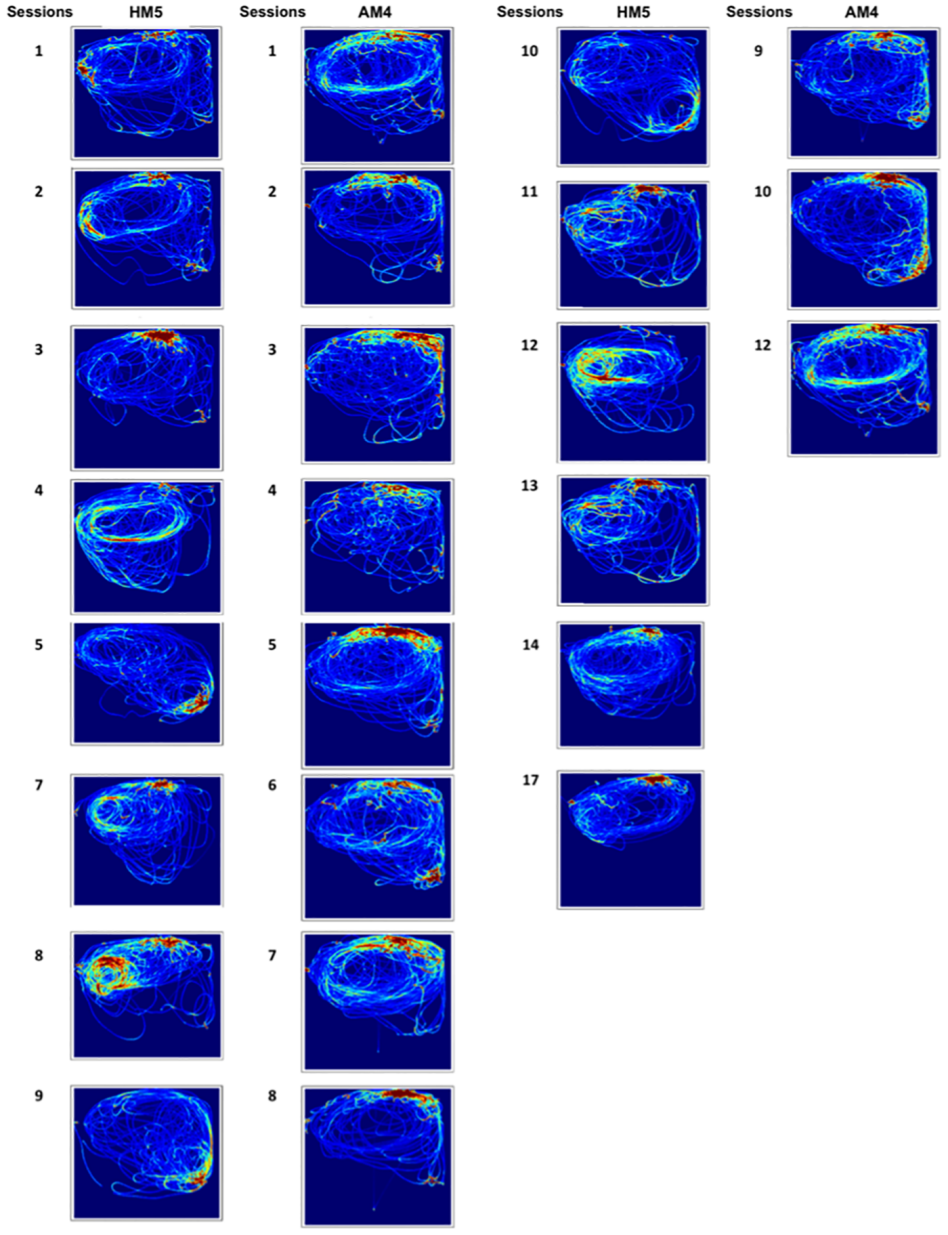
Trajectories of both dolphins throughout the tracking sessions. Red indicates higher occupancy and shows a predominance of circular swimming around the Deep Area.

The 3D trajectories in the pool throughout the sessions were extracted. Fig 2 shows the plots of the tracking sessions for both focal dolphins. As data shows both dolphins use the entire habitat during all sessions, having a predominance of circular swimming in the Deep Area.

To analyse the occupancy patterns of the animals, the frames correspondent to the Deep Area, Shallow Area and Bottom were extracted.

**Fig 3:**
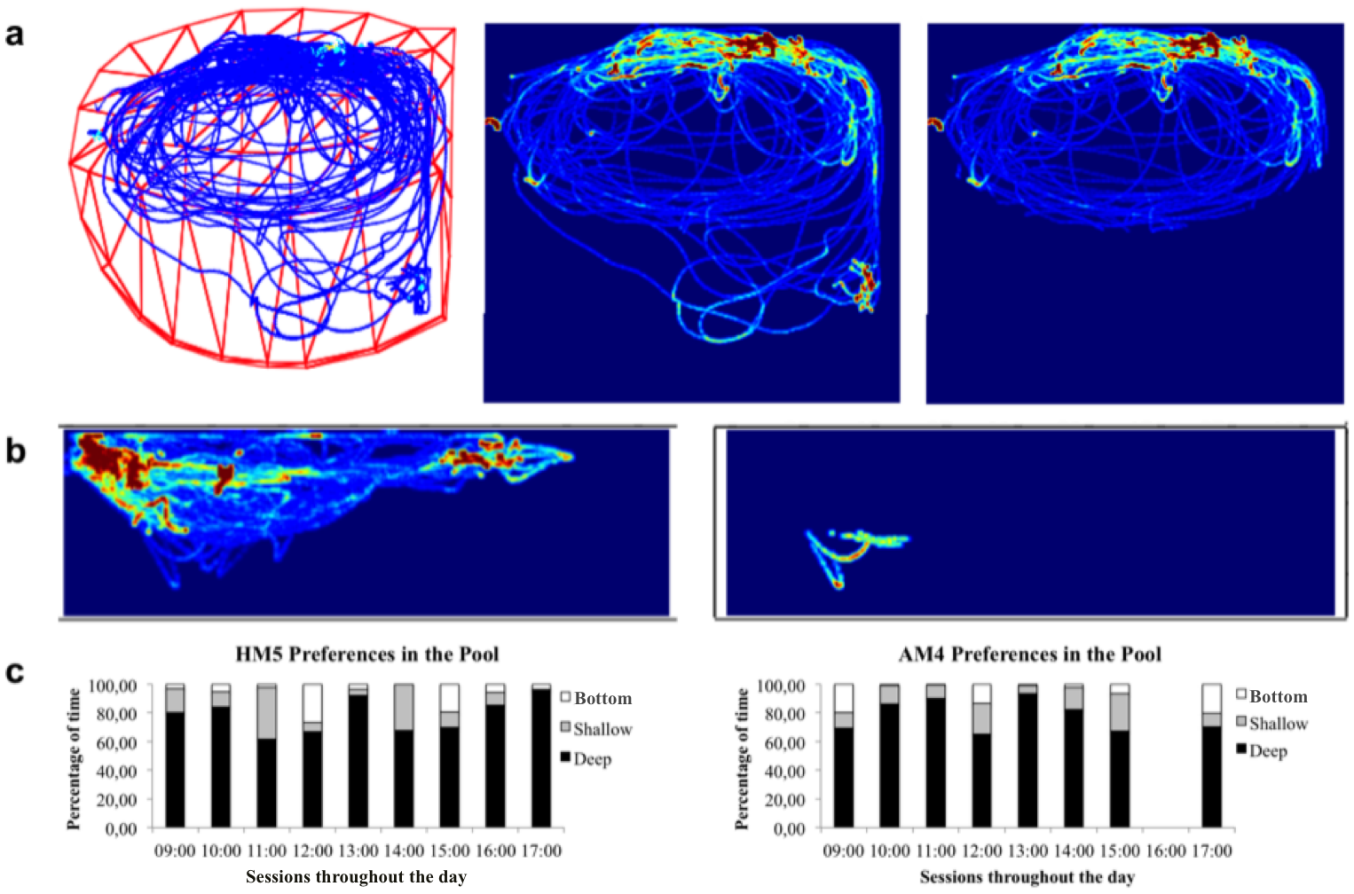
D-Track outputs for dolphin trajectories in the 3D-reconstructed pool. (a) Trajectory of AM4 in one of the sessions with red points representing more time spent in a particular section of the pool and light blue lines representing less occupancy. At the far right, an isolation of AM4’s movements just in the Deep Area of the pool. (b) On the left, the complete path travelled by the animal in one of the sessions, represented laterally, showing a wide 3D occupation of the pool, and on the right a selection of AM4’s path just when the animal was in the Bottom of the pool. (c) Percentage of the time spent by HM5 and AM4 in the Deep, Shallow Areas and Bottom of the pool throughout the day.

HM5 and AM4 seem to present the same occupancy/preferences in the pool. Both dolphins spent more time in the Deep Area (Fig 3a, approximately 85% of the total time (406.85 mins (SD = 9.99 mins) and 444.39 mins (SD = 10.15 mins) for HM5 and AM4, respectively). In relation to the Bottom of the pool, both dolphins spent only around 7% of the non-consecutive 18 hours, about 36.84 minutes (SD = 4.34 mins) and 39.61 minutes (SD = 4.89 mins) for HM5 and AM4 respectively (Fig 3b) (mainly in the 12:00 and 15:00 sessions for HM5 and 09:00, 12:00 and 17:00 sessions for AM4 (Fig 3c).

### Dolphin speeds in the pool

**Fig 4:**
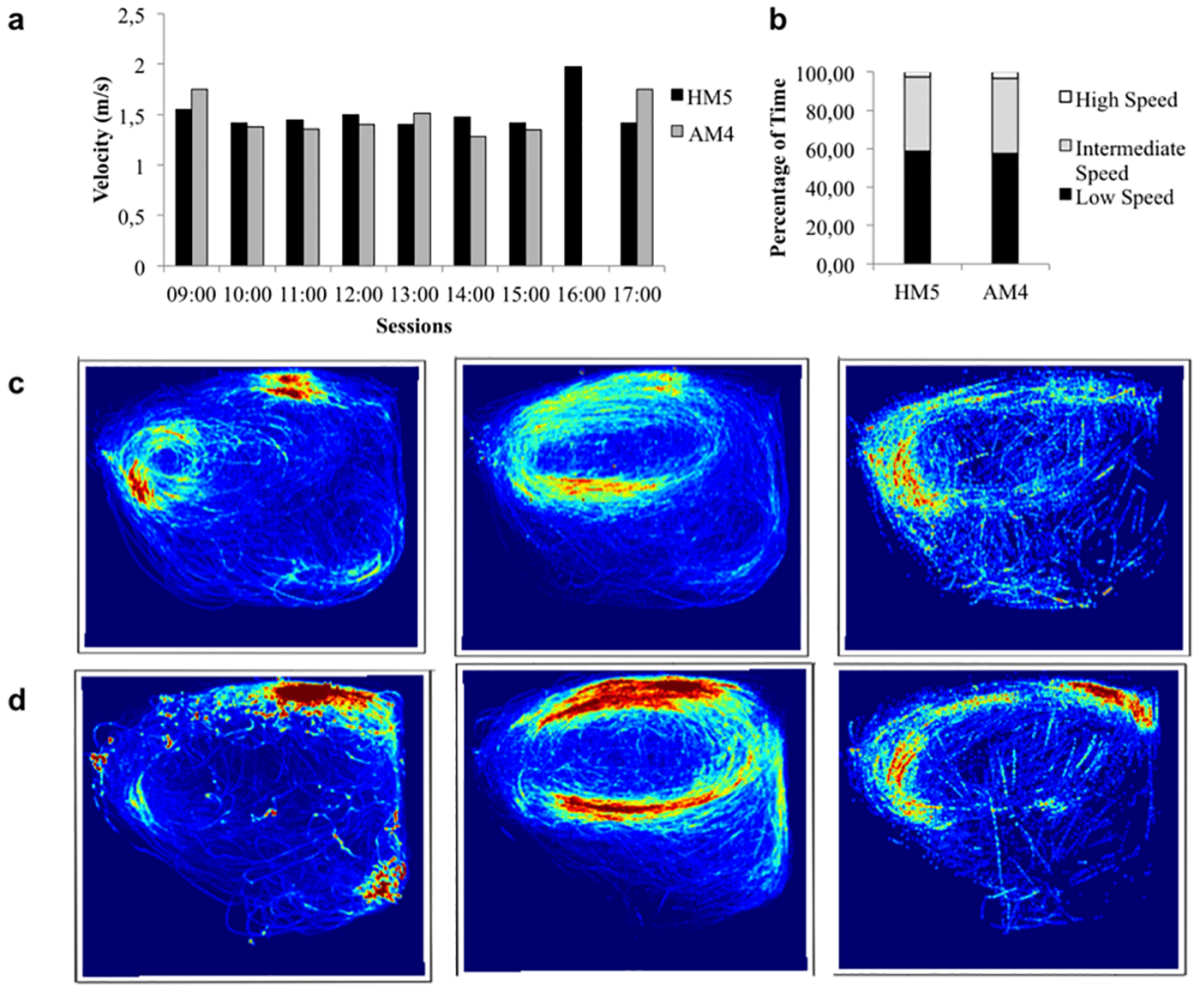
Dolphin speeds for all sessions. (a) Mean speed for HM5 and AM4 for all sessions throughout the day. (b) Percentage of time each dolphin spent in the three speed tertiles throughout all sessions. (c) Speed analysis of the entire data collection for HM5 in the speed tertiles: Low, Intermediated and High speed. The colours represent the occupancy of the animal in the pool, with red points showing more time spent in that particular location at each speed tertile. (d) The same as c) for AM4.

The animals showed very consistent average speeds throughout the day (Fig 4a). Both dolphins spent the same amount of time in the three categories of speed (Fig 4b), and the low speed tertile was the most common. A total of 202.93 minutes (SD = 6.65 mins) corresponding to 58.78% of the total time for HM5, and 244.31 minutes (SD = 3.01 mins) corresponding to 57.73% for AM4. The high speed tertile was the less frequent, with HM5 exhibiting 9.53 minutes (SD = 44.34 secs), corresponding to 2.76% of the total time and AM4 presenting 13.89 minutes (SD = 1.01 mins), corresponding to 3.28%.

Using the trajectory information, we were able to observe where the animal produced lower, intermediate and higher speeds (Fig 4c for HM5 and Fig 4d for AM4). Images show that both dolphins use the three categories of speed through the entire habitat, even though the lower speed category is observed predominantly near the trainer’s location.

### Routines of the animals in the pool

**Fig 5:**
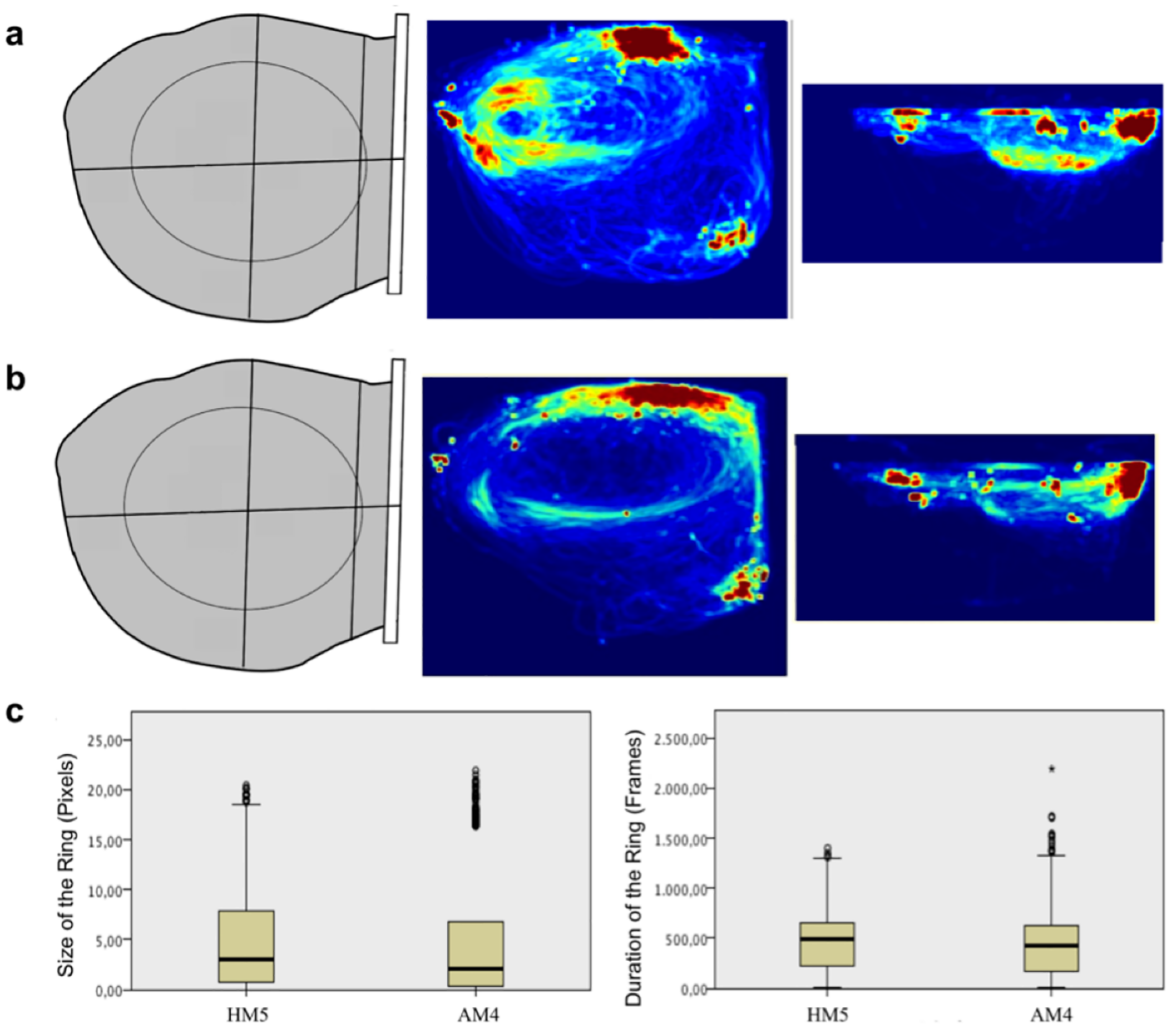
Movement routines of the Dolphins in the pool throughout the sessions. a) Routines of HM5 and b) Routines of AM4 in all sessions, combining the information from the top and lateral views. c) Size and Duration of the swimming rings with significant differences between dolphins (both p < 0.001).

The subjects used the entire partition throughout the sessions, as shown in Fig 5, with no particular preference for the Deep Area.

Analysis of ring duration resulted in a mean number of 469 frames per ring (SD = 303.56 frames), corresponding to 15.63 seconds for HM5, and a mean number of 433 frames per ring (SD = 278.25 frames), corresponding to 14.43 seconds for AM4. This apparent homogeneity hides relevant variation. In fact, the Kruskal-Wallis test revealed significant differences in the size and duration of the rings between animals, (H(1) = 38.570, p < 0.001 (Fig 5c), suggesting individual patterns of circling. It also revealed changes in the size and duration of the rings during individual sessions of HM5 (H(13) = 48.757, p < 0.001 and H(13) = 224.675, p < 0.001, respectively) and AM4 (H(10) = 129.701, p < 0.001 and H(13) = 84.736, p < 0.001, respectively), and between sessions H(1) = 26.162, p < 0.001), supporting the notion of variability in the 3D movement trajectories of the dolphins.

Regarding the possible influence of the trainers’ proximity in the movements of the subjects, data showed that the animals do not spend much time near the trainers’ position, with HM5 spending just 6% of the total time in that area (31.29 mins with SD = 2.29 mins) and AM4 spending around 9% of the time (52.88 mins with SD = 4.42 mins).

As to the possible influences on the subject’s movement patterns of other animals in the adjacent partitions of the pool, the trajectory data showed little time spent near the gates where the other dolphins could be seen by the subjects (5% and 6% of the total time for HM5 and AM4, respectively).

## Discussion

Bottlenose dolphins in captivity are held in habitats that are obviously much smaller and less complex than their natural environments, which inevitably leads to behavioural alterations (as assessed by, e.g. Defran & Pryor [28]). New methods are being developed to improve the quality of life of marine mammals living under human care. It is important to understand all factors that can interfere with the health of animals in captivity, and also to devise ways to monitor and improve their welfare, reducing boredom, stress and stereotyped behaviours. For that, any automatized or semi-automatized system to record and analyse movements over extended periods is highly valuable.

We have optimized a system, labelled D-Track, which allows the semi-automatic video tracking of aquatic animals in a pool, and also performs multiple analyses flexibly. The specific novelty in this development is the ability of tri-dimensional tracking of a large aquatic animal in a wide arena, without the need of a camera above the pool or at a specific angle. The system is adaptable to different artificial settings, and it is easy to implement, requiring only some habitat measurements for the 3D reconstruction of the arena. D-Track simplifies movement analyses that had to be performed manually for years (e.g. Shyan et al. [29]) in a way much more prone to error. This video tracking system is mostly automatic, although some features need to be assigned manually, namely the threshold and some tracking rectifications. Since captive settings for this type of animals are, usually, outdoors, the tracking system must be able to locate the animal with varying light conditions and wind effects, sometimes requiring these manual adjustments. However, it may add small quick jumps in the data (*jittering*), so an outlier’s analysis was carried out. The results confirmed that it is possible to analyse with high detail movement data even with variable light conditions, such as during the sunrise and sunset times of day.

Knowledge of enclosure preferences by the animals, easy to detect with D-Track, allows a better assessment and improvement in their quality of life. It is also useful in the planning of future modifications in the pools or space management.

Results showed that both animals spent about 85% of the time in the Deep Area of the pool, but mostly at the surface, and only 7% in the Bottom, near the 5 meters maximum depth. This is not surprising since these animals have more stimuli at the surface, e.g. the trainers, food and toys. In the Bottom, on the contrary, the animals find only a canvas surface. While in natural environments dolphins may feel motivated to explore deeper, in captivity the animals may prefer to spend less time near the bottom, and that kind of information could be relevant when planning new habitats.

Both dolphins showed a very consistent average speed throughout the sessions, with the slow speed category (up to 1.7 ms^-1^) as the most dominant (58.78% and 57.73%, for HM5 and AM4 respectively). The routine swimming speed of a bottlenose dolphin in the wild is between 1.77 and 3.19 ms^-1^ and the maximum speed registered is 9.7 ms^-1^ (see Goforth’s work [30] and the review of Fish and Hui [31]). Our data shows lower values for the predominant speed (up to 1.7 ms^-1^) and maximum speed (5.1 ms^-^1), which probably reflects the environmental constrains. Since the pool has a diameter of approximately 21 meters, the dolphins may not have motivation to reach higher speeds.

The subjects showed specific movement routines that involved circular swimming over the Deep Area of the pool and used most of their habitat, performing rings with different durations and sizes. These rings presented differences between individuals and were variable across sessions, indicating that these patterns of movement are not highly stereotyped. In any case, having a baseline of the normal movements of captive dolphins facilitates the detection of bizarre behaviours that may emerge. Since stereotyped movements and bizarre behaviours are one of the major welfare concerns of trainers and facilities holding captive animals, such a semi-automatic tracking system may be a very useful resource.

Results showed that the dolphins spent low percentages of time near the trainers or watching other dolphins in adjacent partitions, displaying some degree of autonomy.

Analysis of the routines of captive animals is a time consuming process that may lead to subjectivity and error. Focusing on the needs for behavioural analysis of captive dolphins, we successfully developed a methodology capable of a three-dimensional reconstruction of the artificial habitat and an algorithm that semi-automatically tracks the position of the subject. The maintenance of the D-Track, once adapted to the environment, is simple and straightforward. The flexibility in the camera types and their placements represent a relevant improvement to existing methods. The analysis of trajectories allows the measurement and quantification of the animal’s preferences for pool sections, as well as the detection of stereotypies. This novel application is a user-friendly tool that may be used by institutions interested in measuring or monitoring the behaviour of their dolphins (or other animals). We propose that this non-invasive method is a solution when more intrusive tracking systems, such as subcutaneous transponders or attached data loggers, are not an option.

D-Track is potentially useful to any investigation of motor variability in large aquatic animals, both in intraspecific contexts and in the analysis of interspecific differences.
