# Correction: D-Track—A semi-automatic 3D video-tracking technique to analyse movements and routines of aquatic animals with application to captive dolphins

**DOI:** 10.1371/journal.pone.0211383

**Published:** 2019-01-23

**Authors:** Patrícia Rachinas-Lopes, Ricardo Ribeiro, Manuel E. dos Santos, Rui M. Costa

There is an error in the XML that is causing the third author’s name, Rui M. Costa, to be indexed incorrectly. The name should be indexed as Costa, Rui M. The correct citation is: Rachinas-Lopes P, Ribeiro R, dos Santos ME, Costa RM (2018) D-Track—A semi-automatic 3D video-tracking technique to analyse movements and routines of aquatic animals with application to captive dolphins. PLoS ONE 13(8): e0201614. https://doi.org/10.1371/journal.pone.0201614.

## References

[1] Rachinas-Lopes P, Ribeiro R, dos Santos ME, M. Costa R (2018) D-Track—A semi-automatic 3D video-tracking technique to analyse movements and routines of aquatic animals with application to captive dolphins. PLoS ONE 13(8): e0201614 10.1371/journal.pone.0201614 30114265 PMC6095516

